# Surgery of the Ovaries and Tubes and the Indication Therefor*Read before the North Texas Medical Association, November, 1914.

**Published:** 1915-03

**Authors:** R. L. Hall

**Affiliations:** Italy, Texas


### Surgery of the Ovaries and Tubes and the Indication Therefor.*

BY R. L. HALL, ITALY, TEXAS.

When asked to read a paper on this subject, "Surgery of the Ovaries and Tubes and the Indication Therefor," I accepted, feeling my incompetency to do the subject justice. I hope, however, to say something that may bring out a discussion from which we all may derive some benefit. I believe that there is no subject in the entire domain of surgery in which there is so much difference of opinion as to the proper treatment as there is in the treatment of the ovaries and tubes. Neither do I believe that there is a diseased condition where there is more lack of knowledge of the proper treatment than in the many diseases to which these important organs are subjected. As we are all aware, these delicate organs are caused to bear the brunt of many abuses in these days when criminal abortion is so widespread, both in and out of matrimony. Then there is the ever-present gonorrheal infection to which these organs are subjected, both in the innocent wife and in those who are not so innocent. I believe that under our present social system infection from the causes just mentioned are on the increase. Therefore it behooves us as physicians and surgeons to inform ourselves so that we may be able to apply the best methods for the relief of those who may come to us with their physical and mental suffering. There are too many physicians who depend on internal medicine and local treatment alone for relief, either through a lack of knowledge of the true condition or because they do not feel themselves qualified to perform the proper surgical procedure, and refuse to recommend an operation, thinking

*Read before the North Texas Medical Association, November, 1914.

that it might reflect upon their own ability. By so doing they allow these unfortunate women to seek relief from quack nostrums, faith cures and other worthless sources. As a consequence, many of the afflicted are allowed to remain invalids when they could be relieved by the proper surgical operation. I believe that the general practitioner should so study his cases that he may be able to inform the surgeon to whom he recommends his patient, the probable condition and nature of the operation to be performed, thereby enabling the surgeon to more readily make a proper diagnosis.

If all the general measures and local treatments, such as are outlined in the text-books, do not relieve the patient after a fair trial, the question of an operation should be advised. The operation having been advised and accepted, the surgeon to whom the case is referred, should study the case so as to have a clear idea of what the conditions are, and he should explain to the patient the nature of the operation which is to be performed. He should explain to her the difference between a radical and a so-called conservative procedure. He should tell her that in the larger per cent of cases that nothing short of a radical operation relieves all the symptoms; but that each case must be judged for itself and that he would be as conservative as might be consistent with the best results.

If, when the abdomen is opened, both tubes are found diseased, one being normal except at the abdominal extremity, the former may be removed and the latter resected. If one tube is healthy, it is better to remove the diseased side rather than to do a resection. The relative importance of the ovaries and tubes should always be a great factor in the surgical treatment of these organs. In this treatment our first thought should be the complete restoration of all their functions without pain and to preserve menstruation and ovulation, thereby making conception possible. This, of course, can be done only by removing the diseased parts and leaving all healthy tissue in its place. It is more important to leave all or a part of the ovary than the tube. For, as is well known, upon the ovaries depend ovulation, internal secretion and menstruation. As the sole function of the tubes is to act as transmitters of the ova, and as their removal is not followed by those severe nervous disturbances that may arise from the removal of the ovaries, it is not so important that their function should be preserved.

In case there is pus in the tube, it should be aspirated before an attempt is made to break up the adhesions. In most chronic cases the pus is sterile, so that no harm results from soiling the wound with it. Nevertheless, it is best to remove the tube intact, if possible.

In some cases of pyosalpinx both ovaries are in a fairly normal condition. When such is the case, the tubes alone should be removed. By so doing the menstrual function is preserved and the patient is saved from the discomforts of an artificial menopause. In a great many instances it may be possible to preserve one ovary; in which case a salpingectomy should be done on one side and a salpingo-oophorectomy on the other.

There is never an indication for the removal of the ovaries without the tubes, for when the former are extirpated the latter become useless.

When the ovaries are both involved in the diseased process and it is evident to obtain the best results, both of these structures should be removed. A hysterectomy should always be done, because the uterus becomes absolutely useless after the removal of both ovaries; besides, if left in its place, the endometrium becomes diseased and is very likely to cause a chronic leucorrhea. It is often easier and safer to remove all of the organs en masse than to dissect out both sides separately. Better drainage is always secured by the removal of the uterus.

Malignant diseases of the ovary have always been recognized as constituting a very strong indication for its removal; but for the removal of its fellow as well, when not diseased, is a question for debate. Among the objections urged against the leaving of the ovary, the most important is that of the liability of the disease to recur on the opposite side. I think the age of the patient should be taken into consideration. It should probably be saved in a young woman, with the expectation of carefully watching her for several years and of operating at once upon detecting the slightest evidence of disease.

Ovarian tumors of the connective tissue usually forbid conservatism, as they are apt to involve the entire organ.

There are a great many pathological conditions of the ovaries which are benign where the surgeon is perfectly justifiable in removing the entire ovary. For example: When all of the ovary seems to be taken up by a large hematoma and no sound ovarian tissue can be found, and dermoid cists, especially, when mixed with a cystoma. And it is very rare when any part of the ovary can be saved when there is an ovarian abscess.

Tuberculosis of the tubes is comparatively common. It is stated by our best authorities that it is the cause of about 10 per cent of all cases of salpingitis and pyosalpinx. In a very large proportion of all cases of peritoneal tuberculosis, in women, the apparent focus, so far as the abdominal cavity is concerned, is in the tubes. Unless the disease is very far advanced an operation is indicated. Some are of the opinion that tuberculous tubes should not be removed; but, from my own limited experience and from the knowledge gained from the experience of others, I am convinced that they should be excised, even in the presence of extensive peritoneal involvement. In every case of tuberculous peritonitis, in women, where the abdomen is opened, the tubes should be carefully inspected and if diseased, they should be removed.

I shall not attempt to go into the full details of the operation, but shall endeavor to give a few of the more important parts. As is well known, the removal of the tubes and ovaries is the simplest form of abdominal surgery next to the exploratory incision or the suspension operation for retroflexion of the uterus. Especially is this true where there are no adhesions and are but slightly altered by disease. The structures to be removed, in a simple salpingo-oophorectomy, are the entire length of the tube, the ovary with its hilum and a portion of the utero-ovarian ligament together with their blood-vessels, lymphatics and nerves. The chief risk of the operation, next to sepsis, is hemorrhage from improper control of the blood-vessels. After the ligation is well done by being tied tight, the ovary and tube are removed by cutting the pedicle, at least, one-third of an inch from the ligature. After the appendages have been removed it is better to remove the vermiform appendix and examine the gall-bladder before closing the abdomen. Finally, a careful inspection should be made to determine whether there is any bleeding and to be sure that all sponges, etc., have been removed.

---

From time to time the proprietors of Fellows' Compound Syrup of Hypophosphitis have published interesting information upon the technique of several different Tests, Reactions and Signs. They have received so many letters from busy practitioners who wanted the particulars of the technique that they have compiled a little brochure, in which they have given some of the more recent diagnostic knowledge which has not as yet become generally known. The little brochure entitled "Some Important Memoranda for the Busy Physician," is attractively gotten up, and if you have not already received a copy you may have one for the asking; you will find it very helpful, especially so if you are engaged in private practice.—Ed.

